# Protocooperative Effect of *Sphaerodes mycoparasitica* Biocontrol and Crop Genotypes on FHB Mycotoxin Reduction in Bread and Durum Wheat Grains Intended for Human and Animal Consumption

**DOI:** 10.3390/microorganisms11010159

**Published:** 2023-01-07

**Authors:** Antonia J. Powell, Seon Hwa Kim, Jorge Cordero, Vladimir Vujanovic

**Affiliations:** Department of Food and Bioproduct Sciences, University of Saskatchewan, Saskatoon, SK S7N 5A8, Canada

**Keywords:** Fusarium Head Blight, common/bread wheat, durum wheat, deoxynivalenol, mycotoxins, biocontrol, mycoparasite, *Sphaerodes mycoparasitica*, FHB management, protocooperation, FHB resistance

## Abstract

The occurrence of Fusarium Head Blight (FHB) mycotoxins in wheat grains is a major threat to global food safety and security. Humans and animals are continuously being exposed to *Fusarium* mycotoxins such as deoxynivalenol (DON) and its acetylated derivatives 3ADON and 15ADON through the ingestion of contaminated food or grain-based diet. In this study, a host-specific mycoparasite biocontrol agent (BCA), *Sphaerodes mycoparasitica*, significantly reduced FHB mycotoxin occurrence in harvested wheat grains from *Fusarium graminearum* 3ADON chemotype infected plants in greenhouse. Four genotypes of wheat, two common wheat and two durum wheat cultivars with varying FHB resistance levels were used in this study. Principal Coordinate Analysis (PCoA) using Illumina ITS sequences depicted beta diversity changes in *Fusarium* species indicating that both plant cultivar and BCA treatments influenced the *Fusarium* species structure and mycotoxin occurrence in grains. *Fusarium graminearum* complex (cluster A), *F. avenaceum* and *F. acuminatum* (cluster B), and *F. proliferatum* (cluster C) variants were associated with different FHB mycotoxins based on LC-MS/MS analyses. The predominant FHB mycotoxins measured were DON and its acetylated derivatives 3ADON and 15ADON. The BCA reduced the occurrence of DON in grains of all four cultivars (common wheat: 1000–30,000 µg·kg^−1^.; durum wheat: 600–1000 µg·kg^−1^) to levels below the Limit of Quantification (LOQ) of 16 µg·kg^−1^. A relatively higher concentration of DON was detected in the two common wheat genotypes when compared to the durum wheat genotype; however, the percentage reduction in the wheat genotypes was greater, reaching up to 99% with some *S. mycoparasitica* treatments. Similarly, a higher reduction in DON was measured in susceptible genotypes than in resistant genotypes. This study’s findings underscore the potential of a *Fusarium*-specific *S. mycoparasitica* BCA as a safe and promising alternative that can be used in conjunction with other management practices to minimize FHB mycotoxins in cereal grain, food and feed intended for human and animal consumption.

## 1. Introduction

Fusarium head blight (FHB), or head scab, is the most devastating and widespread disease of small-grain cereals, especially wheat. This ancient plague, with origins in the fertile crescent, is caused by the phytopathogenic *Fusarium graminearum* species complex (FGSC). This FGSC, also known as *F. graminearum* sensu stricto, is a phylogenetically distinct group of 16 taxa that predominates in major wheat-growing areas of North America and other major wheat-producing regions worldwide. [1,2,3]. In wheat, the heads/ears of a growing plant are most susceptible to FHB attack at the beginning of anthesis through the soft dough stage of seed or kernel development [2,4,5]. Following a successful FHB infection, symptomatic and asymptomatic seeds contaminated with mycotoxins may cause a variety of adverse health effects and pose a serious health threat to both humans and livestock [6,7,8]. FHB-associated mycotoxins also play a key role in the disease’s establishment and progression [9]. For instance, *F. graminearum* is a highly virulent hemibiotrophic phytopathogen that produces potent toxins that are known to increase disease severity in wheat under favorable climatic changes [10,11,12,13]. Consequently, FHB and its associated mycotoxins are vital to global agriculture because of the economic burden on the cereal industry, the substantial reduction in grain yield and quality, and the undesirable health effects on humans and animals. Some *Fusarium* mycotoxins, such as deoxynivalenol (DON) and zearalenone (ZEA), are among the most important mycotoxins globally [14]. These two mycotoxins, along with type A trichothecenes, T-2 toxin and HT-2 toxin, and type B trichothecene, and nivalenol (NIV), round out the list of toxicologically important *Fusarium* mycotoxin contaminants in wheat and other cereal crops and food products. [12,13].

The detrimental effects of these *Fusarium*-produced mycotoxins on human and animal health have been well documented [13,15,16,17,18,19]. Type-A trichothecenes such as T-2 toxins are cytotoxic and immunosuppressive. T-2 toxin inhibits protein synthesis and damages lipids. Type B trichothecenes such as DON, also known as vomitoxin, causes intestinal irritation, vomiting, diarrhea, loss of appetite, anorexia, skin dermatitis, necrosis of certain tissue, and feed refusal in animals [16,20]. Trichothecenes also inhibit and disrupt mitotic cell division, and some can also induce apoptosis in normal cells [16]. Zearalenone, also produced by *Fusarium*, is a nonsteroidal estrogenic mycotoxin that affects the functioning of reproductive organs and causes reproductive disorders in pigs and cattle [21]. Therefore, regulatory limits for maximum levels of some FHB mycotoxins in food and feed have been established by various regulating bodies around the world [22,23,24]. The European Commission is one such organization that has set strict legal limits of 1250 ppb for DON in unprocessed common wheat and barley, 1750 ppb for unprocessed durum wheat and oats, 750 ppb for flour, 500 ppb for finished products, and 200 ppb for infant food [22] in an attempt to minimize toxicosis and ensure food safety and security. In both the United States and Canada, the U.S. FDA and Health Canada proposes a maximum limit ~1 ppm/1000 ppb (µg·kg^−1^) for DON in processed food, flour, meal, semolina and flakes derived from wheat [23,24]. Consequently, agricultural and food products with certain *Fusarium* mycotoxins above specified limits are excluded from trade, resulting in significant economic losses. In Canada alone, food loss and waste due to mycotoxin contamination is estimated to be USD 1 billion per year [25,26].

The increasing loss in the wheat industry due to FHB has led to the implementation and practice of several control methods to limit the development and spread of FHB mycotoxins in preharvest cereal crops and postharvest food products. Nevertheless, given the increased *Fusarium* aggressiveness, spectrum of mycotoxins, climate change, lowered pesticide effectiveness and plant resistance, many contemporary FHB management strategies are mostly ineffective and unsustainable [19,27,28,29,30]. Currently, there is no cultivar with complete resistance to FHB in both common wheat and durum wheat. Furthermore, most modern commercially available cultivars possess at best moderate or medium resistance, with the exception of the first ever intermediate-level resistance durum variety, which was developed in AAFC—Swift Current, Saskatchewan Canada—after years of research [31]. Owing to the lack of FHB resistance in modern cultivars against the highly virulent *F. graminearum* 3ADON lines, at present foliar application of fungicides is the most widely used method of controlling FHB and mycotoxins. However, the effectiveness of synthetic FHB fungicides fluctuates based on cultivar type, application time of the fungicide, pathogen sensitivity, health and environmental concerns, agricultural product quality, and cost [6,32]. Compounding this is also an increase in reports globally on *Fusarium* resistance to synthetic fungicide ingredients such as carbendazim, azoxystrobin, and other fungicides used in the management of FHB [33,34,35,36,37]. Additionally, most of the fungicides on the market are formulated to meet to a certain extent the requirements of the first two types of active resistance to FHB as classified by Mesterházy [38]. Type I is resistance against initial infection [39], while type II is resistance to pathogen spread in infected tissue [39]; however, only a few of these synthetic fungicides are able to provide partial resistance to toxins in wheat ears by decomposing them in what is classified as type V resistance [40,41]. Therefore, alternatives that can detoxify, degrade or transform and prevent mycotoxin formation would be an ideal treatment for controlling FHB mycotoxins in wheat and other cereal crops.

Fungal and bacterial biocontrol agents (BCAs) are a group of alterative management strategies that have been shown to produce bioactive compounds that can degrade and detoxify FHB and its associated mycotoxins. These biocontrol agents, some of which are endophytes, live asymptomatically in their host and can provide protection, degrade toxins, and induce the host plant’s immune response against different pathogens [42,43,44,45]. *Trichoderma* and *Clonostachys* (also known as *Gliocladium*) species are well-investigated groups of mycoparasitic generalists which have been extensively used in the agricultural industry as biopesticides and sources of enzymes used in the fight against various plant diseases [45,46,47,48]. A *Fusarium*-specific mycoparasite isolated from wheat fields in Canada, *Sphaerodes mycoparasitica* is a next-generation BCA that has been documented in numerous studies [49,50,51]. *Sphaerodes mycoparasitica* Vujan. is a biotrophic endophyte that not only destroys FHB caused by FGSC (*F. graminearum, F. oxysporum, and F. avenaceum*) species through direct parasitism, but also produces bioactive compounds that can degrade, detoxify, and transform potent toxins into more benign forms. According to Kim and Vujanovic [52], *S. mycoparasitica* substantially reduced DON, 3ADON, 15ADON, and ZEA to less toxic derivatives in a co-culture system. This mycoparasitic BCA also provides prenatal care to wheat plants, protecting germinating seeds from *Fusarium* infestation [50].

Extensive research into fungal endophytes as BCAs of FHB is ongoing [43]; however, there is currently no commercially available biopesticide for common wheat and durum wheat. Even though several studies have already demonstrated the effectiveness of wheat host resistance factors and BCA eco-friendly control, the large-scale implementation of this technology is yet to be realized. Undoubtedly, significant strides have been made using Next-Generation Sequencing (NGS) technology in deciphering the whole genome of common and durum wheat and *F. graminearum*. The transcriptome of *F. graminearum* during the infection of wheat [38] has elucidated different fungal genes coding for metabolites and multiple virulence factors assisting the spread and progression of FHB. However, these insights also highlight the complexity of pathogenic and mycotoxigenic *Fusarium*, suggesting that a more rigorous management system is needed to control FHB and its mycotoxins. In addition, the emergence of new pathogenic *F. graminearum* lineages that have rapidly evolved into more aggressive mycotoxin chemotypes, such as 3ADON compared to 15ADON and NIV, underscores the need for an innovative management strategy. Global warming is another significant factor that makes FHB a greater threat to even the most resistant common and durum wheat cultivars presently on the market. While the most promising cultivars provide a certain level of type I and type II resistance, there is no cultivar that is primed to effectively reduce or minimize the effects of harmful mycotoxins such as DON, NIV, fumonisins (FUM), and aurofusarin (AUS). It is at this juncture that protocooperation between common and durum wheat host genotypes and BCAs specific to aggressive *Fusarium* lineages could be a promising strategy in the management of FHB in cereal crops. 

Protocooperation is an ecological interaction in which two organisms gain benefits through synergy and cooperation. The interaction, however, is not essential to the survival of either organism, as exemplified by the well-known interaction between cattle and egret. There are several studies on *Fusarium*–host (wheat) interaction, host improvement through cultivar optimization, breeding for FHB resistance, fungicide effectiveness, and other management strategies, as revealed by data from the Web of Science Database [53]. However, at the bottom of this FHB management list sits the least explored management area: BCA–*Fusarium*–cereal host interaction. A quick advanced search of the Web of Science database confirms that a great chasm exists between studies that investigate other FHB management strategies and the tripartite interaction between BCA as cereal host supporters or enhancers of resistance against FHB. Indeed, the tripartite *S. mycoparasitica* (BCA)–*F. graminearum* (pathogen)–wheat (plant host) experiments using Synchrotron-based Fourier transform infrared spectroscopy (FTIR) showed that protocooperative strategy is possible; and this BCA can be applied in agriculture fields as an early disease prevention strategy. Nonetheless, there are huge gaps in knowledge pertinent to the domain of protocooperation, such as with respect to (i) BCAs and breeding, (ii) BCAs and cultivars, (iii) BCAs and fungicides, and (iv) BCAs and other management strategies. A search in the Web of Science database also revealed that only 7% of scientific papers on FHB treatment are focused on BCA + breeding and 22% on BCA + cultivars, compared to 71% of papers reporting on BCA with fungicides and other management strategies over the 70-year period (1951–2021) [53]. Hence, there is potential to explore the interaction/protocooperation of BCA and cultivars throughout breeding programs in small-grain cereals as a sustainable alternative to reduce FHB in crops. This area of protocooperation between BCAs and crop genotypes as a possible FHB management strategy is vast, and the possibility for discovering new protective mechanisms using OMICS (genomics–transcriptomics–proteomics) strategies is promising. As the history of controlling FHB in common wheat, durum wheat, and other crops has shown, a more effective resistance against FHB is achieved when management strategies are thoughtfully and carefully combined. This research investigated the effectiveness of the BCA, *S. mycoparasitica* in influencing *Fusarium* species (FGSC) profile, especially *F. graminearum* species complex changes using Illumina sequencing technology and modulating FHB mycotoxins in grains assessed by High-Performance Liquid Chromatography-Tandem Mass Spectrometry (HPLC-MS/MS). The study objectives were (i) to investigate *S. mycoparasitica* effectiveness in reducing FHB mycotoxins in common wheat (*Triticum aestivum*) and durum wheat (*Triticum turgidum* ssp. *durum*) genotypes; and (ii) to investigate its effectiveness in reducing mycotoxins in moderately resistant and moderately susceptible common wheat and durum wheat genotypes.

## 2. Materials and Methods

### 2.1. Greenhouse Trials and Sampling Methods

**Figure 1:**
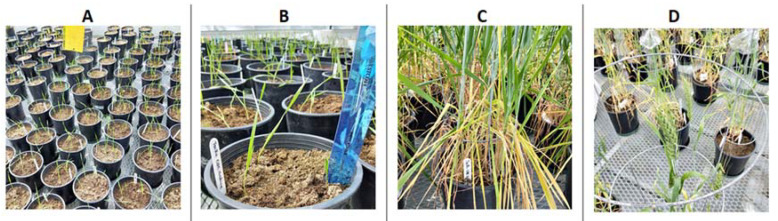
Greenhouse study conducted in Plant Science growth chambers at the University of Saskatchewan. Wheat: (**A**) germinant stage; (**B**) seedling stage; (**C**) vegetative stage with tillers; (**D**) reproductive stage with heads.

All potted common wheat and durum wheat plants were grown in greenhouse (Plant Science and Crop Development Centre (CDC), University of Saskatchewan) growth chambers (Figure 1) separated from those chambers harboring different plant hosts. Seeds (provided by Dr. Hucl of CDC, University of Saskatchewan) were surface disinfected in 75% ethanol for 10 s, rinsed with sterile distilled water for 10 s, submerged for 3 min in 5% sodium hypochlorite (Javex^®^ 12 Bleach, Clorox Company, Spartanburg, SC, USA) and then rinsed five times with sterile distilled water.

There were 10 treatments in total, as described in Table 1. For each treatment, five seeds were placed in one 4 L plastic pot containing 400 g (dry weight) of autoclaved field-capacity Sunshine mix 4 (SunGro Horticulture Canada Ltd., Vancouver, BC, Canada), consisting of three replicates. For treatments in which the BCA was applied at seed, wheat seeds of each cultivar were surface-sterilized and coated with 48-h-grown fungal endophyte SMCD 2220-01 or a combination of SMCD 2220-01 and SMCD 2220-02(5) in treatment 7-SmX_seed_, and then covered with a ~4 cm layer of sterilized Sunshine mix 4 (SunGro Horticulture Canada Ltd., Vancouver, BC, Canada).

In this experiment, the pots were arranged in a randomized complete block design (RCBD) in the greenhouse to mimic the setting in ongoing field trials at the time. Locations of blocks of pots and/or individual pots were changed weekly to account for any variation in the greenhouse conditions. Day and nighttime temperatures varied from 20 to 36 °C and 16 to 20 °C, respectively. Relative humidity during the afternoon and nighttime varied from 50 to 90%. On sunny days, natural sunlight provided irradiation. On cloudy or winter days with reduced daylight photoperiodic conditions, 1000-watt high-pressure sodium lightbulbs supplemented sunlight. These bulbs were suspended from the ceiling roughly 2 m above the plants. Standard-watered plants were kept at about 90–95% water-holding capacity (max. 100% only at the time of watering). A typical application rate of *S. mycoparasitica* to seed as a liquid suspension was 8 g per liter of water as the optimized suspension mix, and 10 mL of that suspension (1 to 2 × 10^6^ CFU-Colony Forming Unit) was used to inoculate a kilogram of seed. This pertains to all treatments in which the BCA was applied to seeds. For treatments in which the BCA was applied at the flowering stage, a typical foliar application range was 8–10 mL BCA + 60 mL water per greenhouse experiment due to the differences in crop size and maturity stage. *Fusarium graminearum* suspensions contained 1 to 2 × 10^4^ CFU and were applied to all treatments except for the control (treatment 1) at the flowering stage during an 8 h window after BCA application. Each treatment for each cultivar had 3 replicates in the greenhouse experiments, giving a total of 120 seed samples. A premium Home & Garden Hand (RL Flo master) sprayer was used to apply inoculants on healthy spikes. After spraying, spikes were covered with transparent plastic bags for 12 h to ensure that the spike was fully contaminated by the inoculant.

### 2.2. Seed Sample Preparation

The harvested plants of each of the 4 cultivars (2 common wheat CDC Go (moderately susceptible—MS) and AAC Brandon (moderately resistant—MR) and 2 durum wheat AAC Strongfield (susceptible—S) and CDC Credence (moderately susceptible plus—MS^+^)) were stored at −18 °C. Before mycotoxin analysis, the plants were removed from storage and the number of spikes on each plant was counted and recorded. Each plant was then threshed spike by spike and the weight of each spike and the weight of grains in each spike were then recorded. The cumulative weight of grains in each wheat plant was recorded and stored in labeled containers. There were three replicates for each treatment (Table 1). In total, 30 grain samples were randomly selected for each cultivar. After the plants in each cultivar and respective treatments were threshed, 15 g of seeds from the 3 replicates for each treatment of each cultivar were packaged, labeled, and analyzed for mycotoxins. There were 10 samples for each cultivar, one for each treatment, giving a total of 40 seed samples less one because no plants were harvested for the CDC Credence cultivar (that showed high sensitivity to the chemical fungicide Prosaro) that was treated with *S. mycoparasitica* + fungicide at the seed (treatment number 3). In all, a total of 39 samples were analyzed.

For Illumina sequencing analysis, only the control (treatment 1) and treatments applied on anthesis Sm_anth_, SmF_anth_., F.gr._anth_ and SmX_anth_ (treatments 4, 5, 6 and 8, respectively) were considered. Approximately 1 g of wheat seeds from each treatment was surface disinfected in 70% ethanol for 30 s, followed by submersion in NaClO (aqueous 1.2% *v*/*v* solution) for 3 min. After the disinfection process, plant material was rinsed 3 times in sterile tap water. Efficacy of surface sterilization was checked by spreading 0.1 mL of the last washes onto 1/10th strength tryptone soy (1/10 TSA) and PDA solidified with 1.5% agar. Next, the surface-disinfected seed material was cut into 0.5 cm portions in aseptic conditions and stored at (−80 °C) until processed for DNA extraction.

### 2.3. Analysis of Fusarium Profile Using High-Throughput Sequencing and Bioinformatics

Total genomic DNA was extracted from seed material using a plant DNA extraction kit (Qiagen Inc., Montréal, QC, Canada). Seed samples (~100 mg) were disrupted using the Precellys 24 Tissue Homogenizer (Bertin Instruments, Montigny-le-Bretonneux, France). DNA extractions were conducted following the manufacturer’s protocols. The DNA yield was quantified using the Qubit DNA HS Assay Kit (Thermo Fisher Scientific, Waltham, MA, USA) and DNA electrophoresis in 1% agarose gels stained with the SYBRTM safe DNA gel stain (Invitrogen, Waltham, MA, USA). DNA samples from replicates of the same treatment were pooled together to an equal DNA ratio. A total of 20 DNA samples were submitted for high-throughput sequencing to the Génome Québec Innovation Centre, McGill University using Illumina MiSeq technology. The PCRs were conducted using the primers ITS1F (5′-CTTGGTCATTTAGAGGAAGTAA-3′) and ITS4 (5′-TCCTCCGCTTATTGATATGC-3′), which amplify the ITS fungal gene [54]. Sample libraries were prepared according to the MiSeq reagent kit preparation guide (Illumina, San Diego, CA, USA), and the sequencing protocol from de la Cuesta-Zuluaga and Escobar, 2016 [55].

Bioinformatics and statistical analyses were performed to portray *Fusarium* diversity in grain samples. ITS sequences derived from extracted DNA from common wheat and durum wheat seeds using high-throughput Illumina technology were analyzed using Mothur version 1.34.3 [56]. The standard operating procedure included the generation of contigs from the combination of forward and reverse reads and the removal of sequence errors and chimeras [57]. Taxonomic classification was performed with naïve Bayesian classifier using the SILVA database. Reads displaying at least 97% identity were clustered into Operational Taxonomic Units (OTUs). After classification, only OTUs corresponding with *Fusarium* spp. were selected for further analysis. The relative abundance of a taxon in a sample was calculated as the percentage of sequence reads belonging to the taxon in relation to the total number of reads in a sample. Principal coordinate analysis (PCoA) based on Bray–Curtis distances was performed using QIIME (Quantitative Insights Into Microbial Ecology) software version 1.9.1 [57] to explore differences in the structure of Fusaria communities between treatments and any relation to the diversity and abundance of FHB mycotoxins (DON, 3ADON and 15ADON). To meet the homogeneity of variance, NGS data were standardized using Hellinger standardization and mycotoxin data were log-transformed. A permutational multivariate analysis of variance (PERMANOVA) was used to test the significant difference between taxonomic fusaria (OTU level) data (beta diversity) and the influence of treatments and plant cultivars using PC-ORD software, Version 7 for Windows [58].

### 2.4. Mycotoxin Analysis by Liquid Chromatography with Tandem Mass Spectroscopy

#### 2.4.1. Sample Preparation

Each grain sample *n* = 39 was first inspected for moisture and size (each particle size had to be <10 mm), without contamination. Samples were then ground with an industrial mill (Ultra Centrifuge Mill ZN 200, Retsch) with a 0.5 mm sieve to obtain samples that were as homogenous as possible. Samples were prepared from 5 g of meal using a direct extraction method for all mycotoxins. The samples were mixed with 20 mL of 80% acetonitrile (ACN)+ 20% water, vortexed and then shaken for 60 min on a mechanical shaker. Following this, the samples were then centrifuged at 3500 rpm for 10 min. One milliliter (1 mL) of the organic phase (supernatant) was then filtered through a 0.45 μm nm PTFE (polytetrafluoroethylene) filter using a syringe. The syringe was triple rinsed with acetonitrile (ACN) between samples and the PTFE filter was replaced between samples. One hundred and sixty microliters (160 μL) of each filtered sample were then added to 40 μL of Internal Standard (ISTD) (this was the same ISTD that was used in the standard-STD curve), which was used to account for the matrix effect. The solution was vortexed and then dried down with nitrogen gas using the Multivap equipment. The resulting sample was then reconstituted in 200 μL of 50/50 MeOH/water solvent. This 200 μL reconstituted sample was then transferred to HPLC vials and centrifuged at 3500 rpm for 10 min. Aflatoxin B1, 3+15ADON (acetyl-deoxynivalenol), deoxynivalenol-DON, diacetoxyscirpenol-DAS, FB1, FB2, HT-2, NIV, ochratoxin A, T-2 toxin, and zearalenone (ZEA) were extracted from ground samples and quantified by UHPLC-MS/MS. The research methodology, separation chromatographs and internal standards for these mycotoxins have previously been published by Nualkaw et al. [59].

#### 2.4.2. Mycotoxin Analysis by LC-MS/MS

The quantification of mycotoxins was performed using a Vanquish UHPLC and TSQ Altis triple quadrupole MS/MS (Thermo Scientific, Mississauga, ON, Canada). The chromatographic column used was a Hypersil GOLD 100 mm × 2.1 mm × 1.9 μm column with a 2.1 mm pre-filter cartridge. The injection volume was 2 μL. The mobile phase consisted of variable mixtures of mobile phase A (LCMS grade water with 0.1% formic acid) and mobile phase B (LCMS grade methanol with 0.1% formic acid) at a flow rate of 0.3 mL/min with a gradient elution program. The total run time was 16 min. The mass spectrometer was operated in both the positive and negative electrospray ionization (ESI) mode. The positive mode analytes were DON and its 3+15ADON derivatives, FB1, FB2, HT2, and T2. The negative mode analytes were NIV and ZEA. The capillary voltage was 3.2 kV for the positive mode and 2.2 kV for the negative mode. Nitrogen was used as the spray gas. Mycotoxins were analyzed using the Selective Reaction Monitoring Mode (SRM). The limit of detection (LOD) was 0.25 μg·kg^−1^ (or 0.25 ppb) for all mycotoxins, while the limit of quantification (LOQ) for DON, 3+15ADON, and NIV was 16 μg·kg^−1^, for FB1 and FB2 were 1 μg·kg^−1^, HT2, T-2, and ZEA were 4 μg·kg^−1^. Mycotoxin analysis was performed by Prairie Diagnostic Services, Saskatoon SK, Canada.

In terms of reagents and chemicals, methanol, acetonitrile, formic acid, and water, all LC-MS grade, were purchased from Fisher, Canada. Mycotoxin standards, 15-acetyl deoxynivalenol (15ADON), 3-acetyldeoxynivalenol (3ADON), aflatoxin B1, Fumonisins B1 (FB1), deoxynivalenol (DON), and diacetoxyscirpenol (DAS) were purchased from Romer, Canada. Mycotoxin internal standards [13C17]-3 acetyl deoxynivalenol, [13C17]-aflatoxin B1, [13C15]-deoxynivalenol, [13C19]-diacetoxyscirpenol, [13C34]-Fumonisins B1, [13C34] fumonisin B2, [13C22]-HT2 toxin, 13C20, ochratoxin A, 13C15-nivalenol, 13C24 T-2 toxin, 13C18, and zearalenone were purchased from Romer, Canada. Stock solutions for all standards were prepared by Prairie Diagnostics, University of Saskatchewan, and stored at −18 °C until analysis. Working standard solution was made by diluting the stock solution in 50% methanol (MeOH) + 50% water. From the individual stock standard solution, a standard mixture was prepared for each mycotoxin.

### 2.5. Statistical Analysis

Statistical analysis for mycotoxins was completed using Statistical Analysis Software (SAS, version 10). All samples with mycotoxin concentration below the LOQ were assigned a value of (LOQ)/2, i.e., 8 μg·kg^−1^ for DON, 3+15ADON, NIV, and DAS, 2 μg·kg^−1^ for T-2, HT-2, and ZEA, and 0.5 μg·kg^−1^ for FB1 and FB2, log_10_ transformed to normalize residuals for analysis of variance (ANOVA).

## 3. Results

### 3.1. Mycotoxins Results

A total of 39 seed samples—CDC Go (*n* = 10), AAC Brandon (*n* = 10), AAC Strongfield (*n* = 10), and CDC Credence (*n* = 9)—were analyzed for 12 mycotoxins—DON, 3ADON and 15ADON, FB1, FB2, NIV, ZEA, T-2 toxin, HT-2 toxin, DAS, ochratoxin A and aflatoxin B1. Of these, nine mycotoxins were quantifiable above their respective LOQs in varying amounts in the samples. Three mycotoxins—aflatoxin B1, DAS, and ochratoxin A—were not quantifiable above their respective LOQs in all samples. Deoxynivalenol (DON) was the major mycotoxin, and was quantifiable in 54% of the samples above its LOQ of 16 μg·kg^−1^, followed by FB1, which was detected in varying amounts in 51% of the samples above the LOQ of 1 μg·kg^−1^, next was 3+15ADON, which was also detected in varying amounts in 13% of the samples above the LOQ of 16 μg·kg^−1^, and FB2 was detected in minimal amounts in 3% of the sample above the LOQ of 1 μg·kg^−1^ (Table 2, Table 3, Table 4 and Table 5). The other mycotoxins—NIV, ZEA, T-2 toxin, and HT-2 toxin—were not detected or were only minimally detected in fewer than 1% of all the samples at levels above their respective LOQs. Table 2, Table 3, Table 4 and Table 5 summarize the concentrations of DON, 3+15ADON, FB1, and FB2 in four cultivars. The major mycotoxins detected in the grain samples were DON, 3+15ADON, Fumonisins B1 and B2 (FB1 and FB2) (Table 4 and Table 5). These four mycotoxins were detected in more than 2% of the grain samples.

### 3.2. Deoxynivalenol (DON) Concentration in Common Wheat and Durum Wheat Cultivars

Deoxynivalenol (DON) was the most frequently detected *Fusarium* mycotoxin and was usually reported as having the highest concentration in the common and durum wheat samples. The highest concentration of DON was seen with treatment 6 (*F. graminearum* applied at anthesis (F.gr._anth._)), as the positive control, in all four cultivars (Table 2). The results of the mycotoxin analysis showed that the BCA-formulated treatments reduced DON concentrations to levels below the LOQ (<16) in 58% of the 31 samples (this number does not include the four samples of the control and the four samples of the F.gr._anth._ treatment). In all of the BCA-treated samples, DON occurrence was significantly reduced by at least 94.2–99.9% across all four cultivars when compared to the *F. graminearum* treatment. Overall, there was a cumulative reduction in DON levels of 99.9% in the CDC Go with all BCA treatments compared to the F.gr._anth._ treatment. For AAC Brandon, there was a cumulative reduction in DON levels of 99% with all BCA treatments compared to the F.gr._anth._ treatment. In both durum cultivars, AAC Strongfield and CDC Credence, there was an overall 97% reduction in DON levels for all BCA treatments when compared to F.gr._anth._ treatments.

The treatments Sm _anth._ and SmF _seed_ + SmF _anth._ were the most effective at reducing the occurrence of DON to levels below the LOQ in all four (4) cultivars compared to both the control and F.gr._anth._ treatments. With these two treatments, the DON levels in both wheat cultivars, CDC Go and AAC Brandon, were reduced by at least 99.9% and 99.8%, respectively, compared to the F.gr._anth._ treatment. In the durum cultivar AAC Strongfield, DON occurrence was reduced by at least 98.4% with both treatments compared to the F.gr._anth._ treatment. In the case of the durum cultivar CDC Credence, Sm _anth._ treatment reduced DON levels by at least 97.2%, and SmF_seed_ + SmF _anth._ treatment reduced DON levels by at least 96.8% when compared to the F.gr._anth._ treatment. In all treatments, the highest DON concentration (29720 μg·kg^−1^) was detected with treatment 6 (F.gr._anth._) in the moderately susceptible wheat cultivar CDC Go (Table 2). The lowest DON levels were detected with treatment 3 (Sm _anth_.) in all four cultivars. Overall, a higher concentration of DON was detected in the two common wheat cultivars (CDC Go and AAC Brandon) compared to the two durum wheat cultivars (AAC Strongfield and CDC Credence. The concentration of DON was also detected as being higher in the two moderately susceptible cultivars (CDC Go and AAC Strongfield) compared to the more resistant cultivars AAC Brandon and CDC Credence (Table 2).

### 3.3. Acetylated 3ADON+15ADON in Common Wheat and Durum Wheat Cultivars

Of the 39 samples that were analyzed for the various mycotoxins, only 13% had 3+15ADON levels above the LOQ of 16 (μg·kg^−1^). The concentration of these mycotoxins was relatively low compared to the DON. These acetylated DON derivatives were only detected in 2% of all samples. Overall, all the BCA treatments reduced the occurrence of 3+15ADON to a significant extent, below the LOQ levels compared to the control and F.gr._anth_. treatments. All eight BCA treatments reduced 3+15ADON levels in CDC Go by at least 96.8% when compared to the F.gr._anth._ treatment. In the other wheat cultivar, AAC Brandon 3+15ADON levels were also significantly reduced by at least 94.3% with all BCA treatments compared to the F.gr._anth_. treatment. There was no comparative reduction in 3+15ADON levels between the BCA treatments and F.gr._anth_. treatment for the cultivar AAC Strongfield. AAC Strongfield was the only cultivar in which 3+15ADON levels were below the LOQ with the F.gr._anth_. treatment. The lowest average of 3+15ADON occurrence in all four cultivars was observed in the moderately susceptible plus cultivar CDC Credence. Only one treatment, F.gr._anth._, induced 3+15ADON occurrence reaching levels above the LOQ. The percentage reduction in 3+15ADON in CDC Credence compared to treatment 6 was at least 26.9%. In AAC Strongfield, the only increase (73.1%) in 3+15ADON levels compared to the control and all other treatments including F.gr._anth._ was recorded with treatment 8 (SmX _anth._). However, the detected amount was way below the regulated amount according to the Canadian Grain Commission monitoring limits.

The highest concentration of 3+15ADON was observed in CDC Go, followed by AAC Brandon, and AAC Strongfield, and the lowest occurrence was found in CDC Credence (Table 3). In general, the two mycotoxins quantified together constituted the third most frequently detected mycotoxin found in all 39 samples. The concentration of these mycotoxins was relatively low compared to the other three frequently detected mycotoxins (DON, FB1, and FB2). The highest concentration of 3+15ADON was seen in the moderately susceptible common wheat cultivar CDC Go (Table 3), while the lowest concentration was seen in the moderately susceptible plus durum wheat cultivar CDC Credence. This was similar to the trend found with the other major mycotoxins. The overall concentration of 3+15ADON was higher in the common wheat cultivars compared to the durum wheat cultivars. The concentration of 3+15ADON was also higher in both moderately susceptible cultivars compared to the more resistant cultivars. There are no legal limits for DON, 3+15 ADON, FB1, and FB2 in Canada; however, there are regulatory guidelines for these mycotoxins in food and feedstuff. None of the samples, except for those that were treated with treatment 6 (F.gr._anth_.) exceeded the World Health Organization guidance values for DON or the recommended tolerance levels for Fumonisins.

### 3.4. Fumonisin B1 (FB1) and Fumonisin B2 (FB2) in Common Wheat and Durum Wheat Cultivars

The occurrence of *Fumonisin* B1 (FB1) and *Fumonisin* B2 (FB2) above LOQ < 1 μg·kg^−1^ was detected in 51.3% and 20.5%, respectively of the 39 seed samples analyzed. FB1 and FB2 were the second and third most frequently detected *Fusarium* mycotoxins in the samples. These two mycotoxins were detected in all four cultivars in varying amounts. The highest occurrence of FB1 and FB2 was recorded with treatments 8 (SmX _seed_) and 9 (*S. mycoparasitica* seed + *S. mycoparasitica* at anthesis (Sm _seed_ + Sm _anth._)) compared to both control and F.gr._anth._ treatments in the moderately susceptible wheat cultivar CDC Go (Table 4 and Table 5). The moderately resistant wheat cultivar AAC Brandon had the second-highest occurrence of FB1 and FB2 with treatments 8 and 9 (Table 4 and Table 5). A marked increase in FB1 and FB2 was also seen in AAC Strongfield with the SmF _seed_ treatment (Table 4 and Table 5). CDC Credence was the only cultivar that had an increase in FB1 and FB2 with treatment 6 (F.gr._anth_). In all of the other three cultivars, the occurrence of FB1 and FB2 was not detected or was slightly above the LOQ of 1. In most of the BCA-treated samples, there was an increase in FB1 levels compared to the control and F.gr_.anth._ treatments. The concentration of FB1 was higher in the common wheat cultivars compared to the durum wheat cultivars, which was also the same trend as that seen for FB2 in the samples. An overall higher concentration of both mycotoxins was seen in the moderately susceptible cultivars compared to the more resistant cultivars of both common and durum wheat (Table 4 and Table 5). In general, the concentration and frequency of FB1 were higher than those of FB2 for all cultivars and treatments.

The highest concentrations of FB2 compared to both the control and F.gr._anth._ treatments were recorded in the wheat cultivars. In comparison to FB2, most of the BCA treatments were effective at reducing or stabilizing the levels of FB2 in all cultivars when compared to the control and F.gr._anth._ Treatments SmF _seed_ and SmF _anth._ were the most effective at reducing or maintaining FB2 levels at below the LOQ in all cultivars compared to both the control and F.gr._anth._ treatments. Overall, in all cultivars, the BCA treatments maintained FB2 levels at or below LOQ < 1 except for a few marked increases in AAC Brandon and CDC Go.

### 3.5. Treatment Effect on Mycotoxin Levels in Common Wheat and Durum Wheat Cultivars

#### 3.5.1. Treatment Effect on DON Level

The data for the effects of the 10 treatments on commonly found mycotoxins in small cereal grains were statistically analyzed with One-Way ANOVA, followed by a post hoc Tukey Test, *p* < 0.05. One-way ANOVA confirmed a significant difference between the BCA treatments and other treatments, and their effects in reducing or exacerbating the different mycotoxins. From the data, F.gr._anth._ (treatment 6) had the greatest effect on DON concentration in all cultivars, resulting in a 100–1000-fold increase in DON concentration relative to the control and the treatments formulated with the BCA *S. mycoparasitica* in each cultivar.

**Figure 2:**
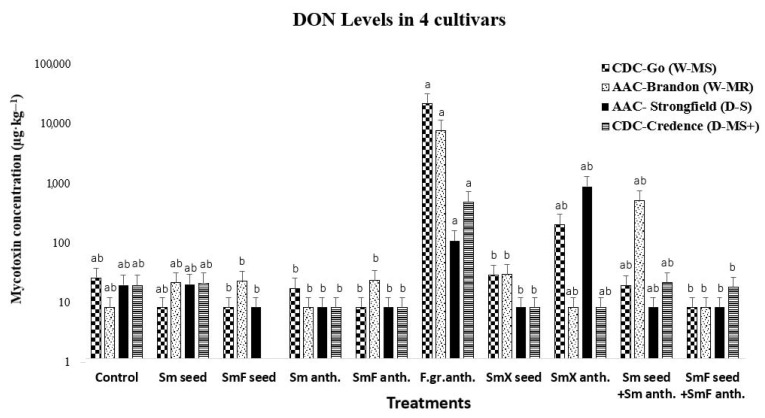
This graph shows the effect of all 10 treatments on DON levels (µg·kg^−1^) in the wheat cultivars CDC Go (moderately susceptible (W-MS)) and AAC Brandon (moderately resistant (W-MR)) and the durum wheat cultivars AAC Strongfield (susceptible (D-S)) and CDC Credence (moderately susceptible plus (D- MS^+^)). The data for each treatment were statistically analyzed using a one-way analysis of variance (ANOVA) with Tukey’s test at *p* = 0.05. Means and standard deviations for each treatment are represented by error bars. The same leters in each treatment are not statistically different. The treatments used were: (1) Control, no biocontrol agents (BCAs) or any other treatment; (2) Sm _seed_-BCA-*S. mycoparasitica* applied on the seed; (3) SmF _seed_-*S. mycoparasitica* + Fungicide on the seed; (4) Sm _anth_.-*S. mycoparasitica* applied at anthesis; (5) SmF _anth._-*S. mycoparasitica* + Fungicide at anthesis; (6) F.gr. _anth_.- a mixture of *F. graminearum* applied at anthesis; (7) SmX _seed_-*S. mycoparasitica* SMCD 2220-01 strain + *S. mycoparasitica* SMCD 2220-02(5) applied on seed; (8) SmX _anth_- *S. mycoparasitica* SMCD 2220-01 strain + *S. mycoparasitica* SMCD 2220-02(5) applied at anthesis; (9) Sm _seed_ + Sm _anth_-*S. mycoparasitica* seed + *S. mycoparasitica* at anthesis; and (10) SmF _seed_ + SmF _anth-_*S. mycoparasitica* + Fungicide seed and *S. mycoparasitica* + Fungicide at anthesis.

In the common wheat cultivars, DON concentration was not quantifiable above the detection level with the Sm _seed_, SmF _seed_, SmF _anth._, or SmF _seed_ + Sm _anth._ treatments, while a slight decrease in DON level was seen with the treatments Sm _anth._ and Sm _seed_ + Sm _anth._ in the moderately susceptible cultivar CDC Go (MS), (Figure 2). For the moderately resistant common wheat cultivar AAC Brandon, most of the treatments had no significant effect on DON levels when compared to the untreated (negative control); however, when compared to the F.gr._anth_. treatment (positive control), there was a significant reduction in DON levels with all BCA treatments. The DON levels in AAC Brandon were the same as in the control with treatments Sm _anth.,_ SmF_seed_, SmF _anth._; however, there was a slight increase in DON concentration with the treatments Sm _seed_ + SmF _anth_. In the durum wheat cultivar AAC Strongfield, DON concentration was not quantifiable with most BCA treatments compared to the control; however, as in the AAC Brandon cultivar, there was a slight increase in DON level with treatment 5—SmF _anth._ In the moderately susceptible plus CDC Credence cultivar, 50% of the treatment showed DON levels below the LOQ or slightly less than the negative control. There was also a slight increase in DON levels with the treatments Sm _seed_, Sm _seed_ + Sm _anth._ in CDC Credence compared to the control. Overall, the highest concentration of DON levels was seen in the common wheat cultivars CDC GO and AAC Brandon, especially with treatments 6–8 in CDC Go and 6, 9–10 in AAC Brandon. One-way ANOVA revealed that the effect of the treatments on DON levels was significant F(9,30) = 3.42, *p* = 0.005. Post hoc analysis using Tukey’s HSD test for multiple comparisons indicated that the effect of treatment F.gr._anth._ was significantly different from the other nine treatments (Figure 2). There were no significant differences between the treatments indicated with the same letter in Figure 2.

#### 3.5.2. Treatment Effect on 3ADON +15ADON 

**Figure 3:**
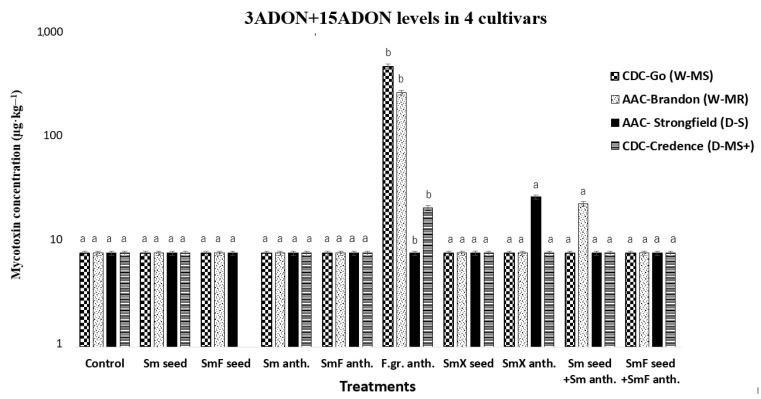
This graph shows the effect of all 10 treatments on 3ADON +15ADON levels (µg·kg^−1^) in wheat cultivars CDC Go (moderately susceptible (W-MS)) and AAC Brandon (moderately resistant (W-MR)) and durum wheat AAC Strongfield (susceptible (D-S)) and CDC Credence (moderately susceptible plus (D-MS^+^)). The data for each treatment were statistically analyzed using a one-way analysis of variance (ANOVA) with Tukey’s test at *p* = 0.05. Means and standard deviations for each treatment are represented by error bars. The same letters in each treatment are not statistically different. The treatments used were: (1) Control, no biocontrol agents (BCAs) or any other treatment; (2) Sm _seed_-BCA-*S. mycoparasitica* applied on the seed; (3) SmF _seed_-*S. mycoparasitica* + Fungicide on the seed; (4) Sm _anth_.-*S. mycoparasitica* applied at anthesis; (5) SmF _anth._-*S. mycoparasitica* + Fungicide at anthesis; (6) F.gr. _anth_.- a mixture of *F. graminearum* applied at anthesis; (7) SmX _seed_-*S. mycoparasitica* SMCD 2220-01 strain + *S. mycoparasitica* SMCD 2220-02(5) applied on seed; (8) SmX _anth_- *S. mycoparasitica* SMCD 2220-01 strain + *S. mycoparasitica* SMCD 2220-02(5) applied at anthesis; (9) Sm _seed_ + Sm _anth_-*S. mycoparasitica* seed + *S. mycoparasitica* at anthesis; and (10) SmF _seed_ + SmF _anth-_*S. mycoparasitica* + Fungicide seed and *S. mycoparasitica* + Fungicide at anthesis.

ANOVA analysis revealed that the effect of the treatments on 3+15ADON levels was significant F(9,30) = 4.05, *p* = 0.002. Post hoc analysis using Tukey’s HSD test for multiple comparisons indicated that the effect of treatment F.gr._anth._ on 3+15ADON concentration in the cultivars was significantly different from all other treatments (Figure 3).

### 3.6. Fusarium Diversity and Associated Accumulation of Mycotoxins in Seeds

**Figure 4:**
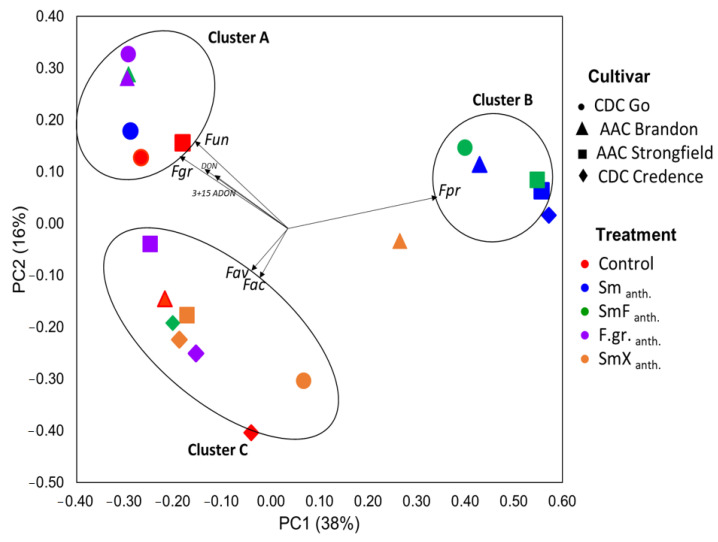
Principal coordinate analysis (PCoA) based on Bray–Curtis dissimilarity between *Fusarium* communities associated with wheat and durum grown in growth chamber conditions. The treatments used were: (1) Control, no biocontrol agents (BCAs) or any other treatment; (4) Sm _anth_.-*S. mycoparasitica* applied at anthesis; (5) SmF _anth._- *S. mycoparasitica* + Fungicide at anthesis; (6) F.gr. _anth_.- a mixture of *F. graminearum* applied at anthesis; (8) SmX _anth_- *S. mycoparasitica* SMCD 2220-01 strain + *S. mycoparasitica* SMCD 2220-02 applied at anthesis. Vectors indicate the correlation between *Fusarium* species (Fac = *F. acuminatum*, Fav = *F. avenaceum*, Fgr = *F. graminearum*, Fpr = *F. proliferatum*, Fun = unclassified *Fusarium* spp.), mycotoxins (DON, 3+15 ADON) and community profiles associated with *S. mycoparasitica* treatments and wheat cultivars.

**Figure 5:**
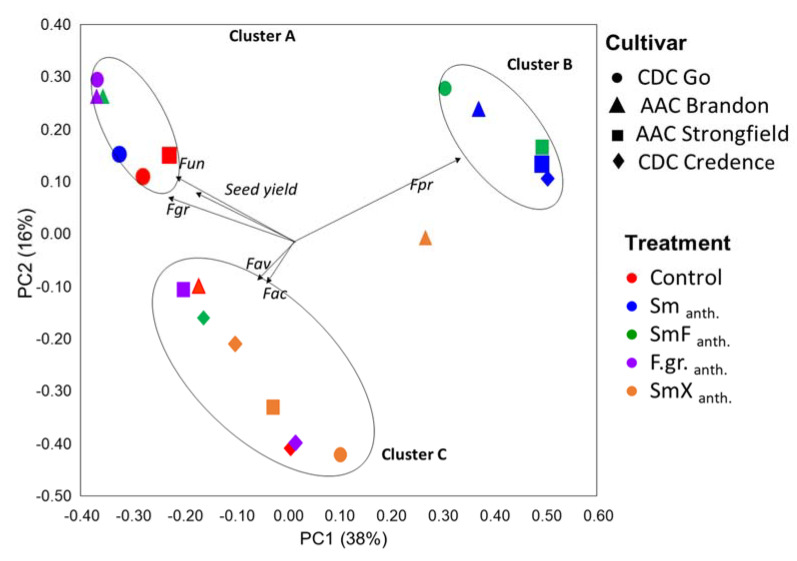
Principal coordinate analysis (PCoA) based on Bray–Curtis dissimilarity between *Fusarium* communities associated with wheat and durum grown in growth chamber conditions. The treatments used were, (1) Control, no biocontrol agents (BCAs) or any other treatment, (4) Sm _anth_. *- S. mycoparasitica* applied at anthesis, (5) SmF _anth._- *S. mycoparasitica* + Fungicide at anthesis, (6) F.gr. _anth_.- a mixture of *F. graminearum* applied at anthesis, (8) SmX _anth_- *S. mycoparasitica* SMCD 2220-01 strain + *S. mycoparasitica* SMCD 2220-02(5) applied at anthesis. Vectors indicate the correlation between *Fusarium* species (Fac = *F. acuminatum*, Fav = *F. avenaceum*, Fgr = *F. graminearum*, Fpr = *F. proliferatum*, Fun = unclassified *Fusarium* spp.), agronomic traits (harvested weight/seed yield) and community profiles associated with *S. mycoparasitica* treatments and wheat cultivars.

Beta diversity of *Fusarium* OTUs, obtained using Illumina ITS sequences, was visualized using PCoA analysis. The results presented in Figure 4 indicate that both plant cultivar and treatment influenced the *Fusarium* community structure and mycotoxin production associated with common wheat and durum wheat seeds. Total variance in the dataset could be explained to an extent of 38% by the first component, and the second component explained another 16%. Ordination results depicted the presence of three clusters corresponding with (A) mostly CDC Go samples and *Fusarium* treatment, SmF _anth._, (B) mostly AAC Strongfield samples with Sm _anth._ and SmF _anth._ treatments, and (C) mostly CDC Credence samples and SmX _anth_ treatment. A positive correlation was detected between *F. graminearum (Fgr)* and *F. unclassified (Fun)* with Cluster A; *F. proliferatum (Fpr)* with Cluster B; and *F. avenaceum (Fav)* and *F. acuminatum (Fac)* with Cluster C. The mycotoxins DON and 3+15ADON were also associated with *F. graminearum* and unclassified Fusarium (*Fun*) within the *F. graminearum* complex in cluster A. No correlation between DON and 3+15ADON was observed with any other Fusarium species of clusters B and C. Permanova analysis indicated a tendency (*p* = 0.08) of F.gr._anth_ treatment effects on *Fusarium* communities and associated mycotoxins (Table 6). As shown in Figure 5, the agronomic trait harvested wheat weight/seed yield was also positively associated with CDC Go and AAC Brandon of Cluster C. Association between AAC Strongfield and harvested seed weight/seed yield was also observed. No correlation was observed between CDC Credence and seed yield, with this possibly being attributable to the low success rate of CDC Credence with F.gr._anth_ treatment. Permanova analysis indicated a tendency of higher effect of BCA (*p* = 0.08) on *Fusarium* communities and associated mycotoxins compared to the cultivar effect (*p* = 0.51); Table 7.

## 4. Discussion

The biocontrol agent (BCA) in this study *Sphaerodes mycoparasitica* was effective at reducing *Fusarium* mycotoxins DON, 3+15ADON, FB1, and FB2 in common and durum wheat under greenhouse conditions. The results of this study are consistent with previous studies on the degradative activity of *S. mycoparasitica* [60]. The level of reduction depended on the time of BCA (*S. mycoparasitica*) application, the plant growth stage, and the treatment, which included BCA only, BCA with other beneficial strains (SmX), and BCA with the fungicide Prosaro. The highest concentration of *S. mycoparasitica* used in this study was 1–2 × 10^6^ CFU/mL, which was an effective treatment dose against *F. graminearum* based on previous in vitro trials (data not shown). The highest concentration of *F. graminearum* used in this study was 1–2 × 10^4^ CFU/mL, which was a mixture of *F. graminearum* 3ADON chemotype SMCD2243 strain and the *F. graminearum* 3ADON, SMCD2910–10B strain. The dosage of Prosaro fungicide used in this study was 50% of the recommended concentration for the Raxil application, as suggested by the manufacturer, Bayer. The fungicide dosage was reduced to (i) highlight the potency of *S. mycoparasitica* in solely degrading pathogenic *Fusarium* mycotoxins under greenhouse conditions, and (ii) assess the effectiveness of reduced chemical fungicide in combination with other management strategies for the treatment of FHB mycotoxins. This study is one of few comparative studies that have so far investigated the effect of the BCA *S. mycoparasitica* on *Fusarium* in common wheat and durum wheat. Even though there are a growing number of studies comparing BCA efficacy in resistant and susceptible common wheat cultivars (biocontrol–cultivar relationship), few to none have examined the efficacy of BCAs in susceptible and resistant durum wheat cultivars. To the best of our knowledge, there is no study reported in the literature that has so far compared the effects of BCA, BCA + fungicide, and BCA–cultivar on FHB in resistant and susceptible common wheat and durum wheat cultivars. 

There is a well-documented relationship between FHB index and DON concentration, as the most frequently detected mycotoxin in wheat and durum globally [61,62,63]. Of the 39 samples that were analyzed in this study, DON occurrence was above the LOQ of 16 µg·kg^−1^ in 54% of the samples. Except for treatment 6 (F.gr._anth._), in which no BCA was added, DON levels detected above the LOQ in these samples were significantly reduced and were considered negligible when compared to the standard and regulations for *Fusarium* mycotoxins in food as set by the Canadian Grain Commission (CGC) [64]. The CGC has established a limit for uncleaned wheat in non-staple foods, which is set at 2000 ppb (2000 µg·kg^−1^); this limit is currently being reviewed [64]. The reduction in DON levels with the eight different BCA treatments confirmed the effectiveness of *S. mycoparasitica* in controlling and reducing the levels of DON in all four cultivars in this study.

In the moderately susceptible cultivar CDC Go, DON was reduced by all BCA treatments by at least 99.9% when compared to F.gr._anth._ (treatment 6). In the moderately resistant wheat cultivar AAC Brandon, there was at least a 90–99% reduction in DON, and in the moderately susceptible plus durum cultivar CDC Credence there was at least a 96–97% reduction in DON. The efficacy of BCAs in reducing DON and other *Fusarium* mycotoxins has been reported in other studies with *Trichoderma-* [47] and *Clonostachys rosea*-formulated CLO-1 [65] products, albeit with limited efficacy. Of all the BCA treatments used in this study, treatment 4 (Sm _anth._) and treatment 9 (SmF _seed_ + Sm F _anth._) were more effective at reducing DON levels in all four cultivars when compared to treatment 6 (F.gr._anth._). These two treatments also reduced DON concentration to comparable levels below that of the control plants. Previous studies on *S. mycoparasitica* [60] revealed that *S. mycoparasitica* degraded DON by 89% to a lesser toxic metabolite, deoxynivalenol sulphate ([M−COCH_3_+SO_3_-CH_2_O]). As was expected, the *Fusarium* treatment F.gr_.anth._ resulted in the highest occurrence of DON in all four cultivars. The *F. graminearum* 3ADON chemotype is a prolific producer of DON, whose occurrence in Canadian common and durum wheat is quite common and is of great concern. DON levels were also higher in both susceptible CDC Go and AAC Strongfield cultivars compared to the two more resistant cultivars. However, DON levels were higher in common wheat cultivars when compared to durum wheat, even though all cultivars received the same dosage of all the inoculants. Durum is the more susceptible of the two varieties, so it would be expected for durum cultivars to have higher levels of DON.

Principal coordinate analysis (PCoA) based on Bray–Curtis distances indicated that both plant cultivar and treatment influenced the *Fusarium* community structure and mycotoxin production associated with common wheat and durum wheat grain. Differences in *Fusarium* diversity profiles in BCA-treated plants were characterized by *F. graminearum* and *F. unclassified* variants associated with cluster A formed by the presence of DON and 3+15ADON. Competitive interactions by non-DON producing natural *F. avenaceum* and *F. acuminatum* under (cluster A) and *F. proliferatum* (cluster C) contaminants were observed. The *Fusarium* species within the B and C clusters are known to prefer different environmental (water and temperature) conditions [66] compared to *F. graminearum* species within the A cluster.

In terms of common and durum wheat’s susceptibility and resistance levels to FHB, our results align with previous findings [67] that compared the reaction of hexaploid and tetraploid wheat to *Fusarium graminearum* chemotypes. It was reported [67] that tetraploid genotypes inoculated with the 3ADON chemotype showed lower disease symptoms compared to hexaploid genotypes, suggesting that the difference in pathogenicity of each chemotype may be due to the different resistance mechanisms of each genotype to the chemotypes and their associated mycotoxins. In our study, the *F. graminearum* 3ADON chemotype was used as the *Fusarium* inoculant. All plants under BCA treatment were co-infected with *Fusarium graminearum* 3ADON except for the untreated plants (negative control). Most BCA treatments applied on these plants reduced DON concentration in grains below the detectable LOQ < 16 ppb (parts per billion) level compared to individual *F. graminearum* 3ADON application (F.gr._anth._—positive control) without BCA co-inoculation, which resulted in a DON concentration of 10,000–30,000 ppb in common wheat and a concentration of 500–1000 ppb in durum wheat cultivars. For instance, the positive control F.gr._anth._ was applied at the same rate, same concentration, and in the same amount to all wheat and durum cultivars, yet in CDC Go, the DON concentration was thirty times greater than the DON concentration in AAC Strongfield, while the DON concentration in AAC Brandon was ten times greater than the DON concentration in CDC Credence. Currently, there is no research or evidence reported in the literature that explains the mechanism underlying the differential responses of wheat genotype to different chemotypes of *Fusarium* species. However, this study, along with other previous work, postulates that the difference in resistance among genotypes may be related to the differential degradation of mycotoxins produced by the 3ADON and 15ADON chemotypes. Walkowiak et al. [68,69] found variation in the genomes of 3ADON and 15ADON chemotypes using a comparative genomics approach. It was found that the difference in genotypes between these two chemotypes could potentially explain the difference in their aggressiveness and the interaction with host plants that results in diverse levels of disease symptoms and DON production. Despite the difference in DON concentration in common wheat and durum wheat genotypes, *S. mycoparasitica* significantly reduced DON levels in all four genotypes relative to that of their respective control. In the wheat cultivars, there was an average 99.5% reduction of DON concentration with all BCA formulations, while in durum wheat cultivars, there was an average 97% reduction with all BCA treatments. The most effective treatments were SmX _anth._ and SmF _seed_ + SmF _anth._ These two treatments reduced DON to undetectable levels in all genotypes when compared to the control (untreated seeds). For 3+15ADON, all eight *S. mycoparasitica* treatments except for one in the wheat cultivar AAC Brandon significantly reduced these mycotoxins to undetectable levels in all four genotypes. Overall, all BCA treatments resulted in reduced DON-vomitoxin concentrations in grains to levels considered negligible when compared to the 1000–2000 ppb (part per billion/µg·kg^−1^) limit based on regulations for *Fusarium* mycotoxins in foods set by the U.S. FDA and Health Canada.

In this study, the effects of the BCA *S. mycoparasitica* on the acetylated derivatives of DON, 3+15ADON, were also investigated. These two mycotoxins are sometimes separately quantified, but in this study, they were grouped as one. Separating these isomers can be tedious and complex, and it is possible that the chromatographic column used in the separation of these *Fusarium* mycotoxins was not efficient in separating the two isomers. Both 3ADON and 15ADON are position isomers that differ in the presence or absence of acetyl groups at C-3 and C-15, [70], 3ADON has a C-3 acetyl group but lacks a C-15 acetyl, whereas 15ADON has a C-15 acetyl but lacks a C-3 acetyl. These isomers are precursors of DON, with either 3ADON or 15ADON being more aggressive in producing elevated levels of DON depending on location and climatic conditions [71,72]. Therefore, it is useful to know which isomer is produced by *F. graminearum*.

The occurrence of 3+15ADON in both common and durum wheat was relatively low compared to the occurrence of DON in the same samples. These mycotoxins were only detected above the LOQ of 16 µg·kg^−1^ in five of the 39 samples; three of these samples were treated with F.gr._anth_. (treatment 6), one sample was treated with Sm _seed_ + Sm _anth_. (treatment 9) in the common wheat cultivar AAC Brandon, and the other sample was treated with SmX _anth_. (treatment 8) in the durum wheat cultivar AAC Strongfield. The levels of 3+15ADON detected in the two BCA-treated samples were relatively low compared to the control, 23.8 µg·kg^−1^ for AAC Brandon and 27.7 µg·kg^−1^ for AAC Strongfield (Table 3). Currently, there are no set limits for 3+15ADON levels in food or feeds. The Canadian Food Inspection Agency has a monitoring program for mycotoxin in livestock feed, but no limits have been set. Both isomers have been shown to have different adverse effects on mammals [73]. With the exception of these two samples, the BCA treatments resulted in at least a 96.8% reduction of 3+15ADON levels in CDC Go, a 94% reduction in AAC Brandon, and at least a 28% reduction in CDC Credence. There was no comparable reduction for AAC Strongfield and the F.gr._anth._ treatment, since 3+15ADON levels were not detected above the LOQ of 16 for the F.gr_.anth._ treatment. In all BCA treatments with AAC Strongfield, except treatment 7 (SmX _anth._), 3+15ADON levels were below the LOQ. Overall, 3+15ADON levels were higher in common wheat cultivars than in durum wheat cultivars and the BCA was efficient in reducing or maintaining 3+15ADON levels, as shown in Figure 3.

Fumonisins (FUMs), an atypical group of mycotoxins in wheat, were also detected in this study. However, the maximum < 300 ppb concentration of FB1+FB2 detected in common wheat and <200ppb in durum wheat grain was negligible, considering that <2000 ppb of total FUMs (FB1 + FB2 + FB3) in food and 5000 ppb in feed are considered safe by the U.S. Food and Drug Administration Agency (2018). Fumonisins are usually produced by different species, including *F. proliferatum* and *F. verticilloides* (syn. *F moniliforme*), in maize. This mycotoxin group includes 28 different forms of Fumonisins, which are designated as being either A, B, C, or D series. FB1 is the most common and economically important, followed by FB2 [74]. In this study, FB1 and FB2 were detected above the LOQ of 1 in all four cultivars with all BCA treatments at least once. The occurrence of FB1 and, to a lesser extent, FB2 in most of the BCA-treated samples was greater than that of the occurrence of FB1 and FB2 with F.gr._anth_. treatment. The only BCA treatment that was effective in reducing FB1 in all four cultivars to levels comparable with the control and F.gr._anth._ treatments was Sm _anth_. FB1 and FB2 levels were significant in the moderately susceptible plus durum wheat cultivar CDC Credence with treatment 6—F.gr._anth._—in reference to the other treatments, while there was a slight increase in FB2 with the same treatment in the moderately susceptible common wheat cultivar CDC Go. It can be theorized that the increase in FB1 and FB2 in these cultivars, as well as with the other BCA treatments, is based on a competition for nutrients and space between the different *Fusarium* species. *Fusarium graminearum*, which produces DON, and *F. proliferatum*, which produces Fumonisins, are competing species, of which *F. graminearum* is the most aggressive. In the absence of effective control, *F. graminearum* dominates, and the probability of DON production increases, which is evident in the F.gr._anth_. treatment. However, in the presence of an effective biocontrol such as the biotrophic *S. mycoparasitica*, *F. graminearum* growth is controlled, and its mycotoxins degraded, as reflected in Table 2 and Table 3. As a result of this, an opportunistic fungal parasite such as *F. proliferatum* has little or no competition for the host’s resources, and as such is free to proliferate and produce the Fumonisin toxins, as seen with the BCA-treated samples in Table 4 and Table 5. This might explain why in three cultivars, CDC Go, AAC Brandon, and AAC Strongfield, the level of Fumonisins was below the LOQ with F.gr._anth._ However, in the cultivar, CDC Credence, the levels of FB1 and B2 increased only with F.gr._anth._ treatment. There is also the possibility that prolonged seed storage under cool (4 °C) conditions and reduced seed moisture prior to mycotoxins analysis could have created conditions conducive to mycotoxigenic *Fusarium proliferatum* and *F. moniliformae* proliferation in seeds outside the greenhouse experiments. The continuous growth of *F. proliferatum* and *F. moniliformae* FB1/FB2 strains may specifically occur in seeds at cool (4 °C) storage temperature and under reduced moisture a_w_ (0.994–0.96) conditions [74]. The minimal temperatures for *F*. *graminearum* activity were situated between 7 to 10 °C [75] which is similar to *S. mycoparasitica*, indicating a possibility of the fungal latency stage in seed under prolonged incubation at 4 °C. Even though the concentration of Fumonisins detected in the greenhouse samples was negligible compared to current allowances; this finding is important, because over the last decade there has been an increase in reports of FB1 and FB2 occurrence in food and food products globally.

In Canada, research on mycotoxin incidence in maize-, oat-, wheat-, and rice-based cereals was performed using samples from Canadian retailer marketplace over 3 years [70]. In that report, Fumonisins were detected in 17% (5/29) of wheat-based samples with a mean occurrence of 3 ppb. The natural occurrence of Fumonisins in common wheat and durum wheat was also detected in crops in Argentina, the US, Europe, Africa, and Asia [76]. Guo et al. [77] reported that *F. proliferatum* strains, producers of FB1 and FB2 in wheat originating from different hosts were able to infect wheat via seed (systemic colonization), leading to the accumulation of Fumonisins in kernels. The level of FB1 occurrence in the wheat kernels was much lower than the levels commonly found in maize. Cendoya et al. [78] also explained that water activity—a_w_ (0.995–0.90)—and temperature also affect the growth and production of fumonisin in wheat by *F. proliferatum*. The same study also reported that because *Fusarium* species may be present in a substrate for extended periods during which a_w_ may change, it is important to know both the optimal and suboptimal a_w_ ranges for growth. In this study, the experiment was conducted under greenhouse conditions. The harvested seeds were stored for 2 years at 4 °C. If Fumonisins contamination occurs via systemic colonization and current research does not suggest stem to seed transfer, then the transfer from seed to plant and back to seed would have to be ruled out, leaving the question of how Fumonisin contamination occurred in these samples unanswered. To answer this question, more research is needed, which is beyond the scope and purpose of this study. Nevertheless, the detection of Fumonisins in common and durum wheat in this study is in agreement with the findings of previous research. Fumonisin concentration was higher in common wheat compared to durum wheat. In wheat, FB1 concentration ranged from <1~338 ppb, (>100 ppb measured in 4/39 samples assessed), and 164 ppb was measured in only 1/20 samples assessed. FB2 concentration in most of the 39 samples was <1 ppb, while only one sample showed 137.7 ppb concentration in durum. Although a negligible amount of FB1+FB2 was detected in this study’s samples based on globally accepted (<2000 ppb/food and <5000 ppb/feed) standards, it is imperative to continue monitoring their occurrence in grain and grain-based food and feed. The lowest concentration of Fumonisins in each genotype was observed with SmX _seed_-BCA treatment as promising solution to efficiently control these dangerous mycotoxins in grain-based diet for human and animal consumption.

## 5. Conclusions

This study confirms the efficacy of the BCA *Sphaerodes mycoparasitica* in shaping mycotoxigenic *Fusarium* diversity and community structure in grains of common and durum wheat cultivars. BCA significantly reduced FHB mycotoxins DON, 3ADON+15ADON, FB1, and FB2 in plants infected with *F. graminearum* under greenhouse conditions. From the results of this study, it was evident that *S. mycoparasitica* applied at the flowering stage was more effective in controlling and degrading the above-mentioned mycotoxins. All eight BCA treatments were effective in reducing all FHB-associated mycotoxins by at least 80–99% across all cultivars in comparison to the F.gr._anth._ treatment. Some BCA treatments were more effective in common wheat compared to durum wheat. The SmF _anth._ and Sm _anth._ treatments were effective in reducing all mycotoxins when compared to both the control and F.gr._anth._ treatments, while the treatments SmF _anth._ and SmF _seed_ + SmF _anth._ were more effective in the common wheat cultivars. Overall, Sm _anth._ was the most effective treatment in reducing all mycotoxins, even to levels below that of the control. The concentration of all mycotoxins was higher in common wheat than in durum wheat, as well as in susceptible cultivars compared to the more resistant cultivars. The efficacy of *S. mycoparasitica* in both common and durum wheat is similar; however, in terms of percentage reduction of mycotoxins, the BCA seems to be more effective in common wheat cultivars. These results also suggest that genetic FHB resistance vs. susceptibility in wheat varieties is an important contributor to the reduction or elevation of DON levels respectively, and our ongoing field studies are designed to answer this question.

## Figures and Tables

**Table 1:** List of treatments.

Treatment	Description
1. Control	No biocontrol agents (BCAs) or any other treatment
2. Sm_seed_	BCA- *S. mycoparasitica* 2220-01 (main BCA strain used in all studies) applied on seed
3. SmF_seed_	*S. mycoparasitica* 2220-01 + Fungicide on seed (1/2 of the effective dose of fungicide- Prosaro was used)
4. Sm_anth._	*S. mycoparasitica* 2220-01 applied at anthesis
5. SmF_anth._	*S. mycoparasitica* 2220-01+ Fungicide at anthesis
6. F.gr_anth_	A mixture of *F. graminearum* 3ADON strains applied at anthesis
7. SmX_seed_	*S. mycoparasitica* SMCD 2220-01+ SMCD 2220-02(5) mixture of BCA beneficial strains, applied on seed
8. SmX_anth._	*S. mycoparasitica* SMCD 2220-01 + SMCD 2220-02(5) applied at anthesis
9.Sm_seed_ + Sm_anth._	*S. mycoparasitica* 2220-01 seed + *S.mycoparasitica* 2220-01 at anthesis
10. SmF_seed_ + SmF_anth._	*S. mycoparasitica* 2220-01 + Fungicide seed and *S. mycoparasitica* 2220-01+ Fungicide at anthesis.

All plants in all treatments except for control (treatment 1) and *F. graminearum* (treatment 6) were inoculated with *F. graminearum* after the BCA formulation was applied. The Fusarium inoculant was added during an 8 h window after BCA application.

**Table 2:** Effect of BCA on DON concentration (µg·kg^−1^) in four wheat cultivars.

	Treatments	CDC Go W-MS	AAC Brandon W-MR	AAC Strongfield D-S	CDC Credence D-MS^+^
1	Control	26	<16	19.8	19.5
2	Sm _seed_	<16	21.7	20.2	21.4
3	SmF _seed_	<16	23.1	<16	NR
4	Sm _anth._	17.2	<16	<16	<16
5	SmF _anth._	<16	23.9	<16	<16
6	F.gr._anth._	29720	10150	1052	564.9
7	SmX _seed_	29.4	30	<16	<16
8	SmX _anth._	227.5	<16	116.7	<16
9	Sm _seed_ + Sm _anth._	19.3	592.3	<16	21.7
10	SmF _seed_ + SmF _anth._	<16	<16	<16	17.9
	Total DON conc. in each cultivar	30071.4	10873	1256.7	677.4

All plants in all treatments except for the control were inoculated with *F. graminearum* within an 8 h window after the application of the BCA treatment. Concentrations at the Limit of Quantification LOQ < 16 were first divided by 2 for an approximate value, which is used in the calculation of total DON concentration (µg·kg^−1^). The treatments used were: (1) Control, no biocontrol agents (BCAs) or any other treatment; (2) Sm _seed_-BCA-*S. mycoparasitica* applied on the seed; (3) SmF _seed_-*S. mycoparasitica* + Fungicide on the seed; (4) Sm _anth_.-*S. mycoparasitica* applied at anthesis; (5) SmF _anth._-*S. mycoparasitica* + Fungicide at anthesis; (6) F.gr. _anth_.- a mixture of *F. graminearum* applied at anthesis; (7) SmX _seed_-*S. mycoparasitica* SMCD 2220-01 strain + *S. mycoparasitica* SMCD 2220-02(5) applied on seed; (8) SmX _anth_- *S. mycoparasitica* SMCD 2220-01 strain + *S. mycoparasitica* SMCD 2220-02(5) applied at anthesis; (9) Sm _seed_ + Sm _anth_-*S. mycoparasitica* seed + *S. mycoparasitica* at anthesis; and (10) SmF _seed_ + SmF _anth-_*S. mycoparasitica* + Fungicide seed and *S. mycoparasitica* + Fungicide at anthesis.

**Table 3:** Effect of BCA on 3ADON+15ADON concentration (µg·kg^−1^) in four wheat cultivars.

	Treatments	CDC Go W-MS	AAC Brandon W-MR	AAC Strongfield D-S	CDC Credence D-MS^+^
1	Control	<16	<16	<16	<16
2	Sm _seed_	<16	<16	<16	<16
3	SmF _seed_	<16	<16	<16	NR
4	Sm _anth._	<16	<16	<16	<16
5	SmF _anth_.	<16	<16	<16	<16
6	F.gr. _anth._	499.4	278.8	<16	21.9
7	SmX _seed_	<16	<16	<16	<16
8	SmX _anth._	<16	<16	27.7	<16
9	Sm _seed_ + Sm _anth._	<16	23.8	<16	<16
10	SmF _seed_ + SmF _anth._	<16	<16	<16	<16
	Total 3 + 15ADON conc. in each cultivar	571.4	366.6	99.7	85.9

All plants in all treatments except for the control were inoculated with *F. graminearum* within an 8 h window after the application of the BCA treatment. Concentrations at the Limit of Quantification LOQ < 16 were first divided by 2 for an approximate value, which were used in the calculation of total 3+15ADON concentration (µg·kg^−1^). The treatments used were: (1) Control, no biocontrol agents (BCAs) or any other treatment; (2) Sm _seed_-BCA-*S. mycoparasitica* applied on the seed; (3) SmF _seed_-*S. mycoparasitica* + Fungicide on the seed; (4) Sm _anth_.-*S. mycoparasitica* applied at anthesis; (5) SmF _anth._-*S. mycoparasitica* + Fungicide at anthesis; (6) F.gr. _anth_.- a mixture of *F. graminearum* applied at anthesis; (7) SmX _seed_-*S. mycoparasitica* SMCD 2220-01 strain + *S. mycoparasitica* SMCD 2220-02(5) applied on seed; (8) SmX _anth_- *S. mycoparasitica* SMCD 2220-01 strain + *S. mycoparasitica* SMCD 2220-02(5) applied at anthesis; (9) Sm _seed_ + Sm _anth_-*S. mycoparasitica* seed + *S. mycoparasitica* at anthesis; and (10) SmF _seed_ + SmF _anth-_*S. mycoparasitica* + Fungicide seed and *S. mycoparasitica* + Fungicide at anthesis.

**Table 4:** Effect of BCA on FB1 concentration (µg·kg^−1^) in four wheat cultivars.

	Treatments	CDC Go W-MS	AAC Brandon W-MR	AAC Strongfield D-S	CDC Credence D-MS^+^
1	Control	<1	<1	2.9	<1
2	Sm _seed_	2.03	4.8	<1	3.9
3	SmF _seed_	22.8	<1	163.5	NR
4	Sm _anth._	<1	<1	2.4	<1
5	SmF _anth._	4.13	25.3	<1	<1
6	F.gr._anth._	2.1	<1	<1	23.2
7	SmX _seed_	338	<1	7.79	<1
8	SmX _anth._	2.5	11.2	<1	8.11
9	Sm _seed_ + Sm _anth._	5.4	268.1	<1	<1
10	SmF _seed_ + SmF _anth._	<1	163.6	42.1	<1
	Total FB1 conc. in each cultivar	378.5	475.5	224.2	38.21

All plants in all treatments except for the control were inoculated with *F. graminearum* within an 8 h window after the application of the BCA treatment. Concentrations at the Limit of Quantification LOQ < 16 were first divided by 2 for an approximate value, which was used in the calculation of total FB1 concentration (µg·kg^−1^). The treatments used were: (1) Control, no biocontrol agents (BCAs) or any other treatment; (2) Sm _seed_-BCA-*S. mycoparasitica* applied on the seed; (3) SmF _seed_-*S. mycoparasitica* + Fungicide on the seed; (4) Sm _anth_.-*S. mycoparasitica* applied at anthesis; (5) SmF _anth._-*S. mycoparasitica* + Fungicide at anthesis; (6) F.gr. _anth_.- a mixture of *F. graminearum* applied at anthesis; (7) SmX _seed_-*S. mycoparasitica* SMCD 2220-01 strain + *S. mycoparasitica* SMCD 2220-02(5) applied on seed; (8) SmX _anth_- *S. mycoparasitica* SMCD 2220-01 strain + *S. mycoparasitica* SMCD 2220-02(5) applied at anthesis; (9) Sm _seed_ + Sm _anth_-*S. mycoparasitica* seed + *S. mycoparasitica* at anthesis; and (10) SmF _seed_ + SmF _anth-_*S. mycoparasitica* + Fungicide seed and *S. mycoparasitica* + Fungicide at anthesis.

**Table 5:** Effect of BCA on FB2 concentration (µg·kg^−1^) in four wheat cultivars.

	Treatments	CDC Go W-MS	AAC Brandon W-MR	AAC Strongfield D-S	CDC Credence D-S^+^
1	Control	<1	<1	<1	<1
2	Sm _seed_	<1	<1	<1	<1
3	SmF _seed_	2.3	<1	11.8	NR
4	Sm _anth._	<1	<1	<1	<1
5	SmF _anth._	<1	4.04	<1	<1
6	F. gr._anth._	<1	<1	<1	4.1
7	SmX _seed_	137.7	<1	<1	1
8	SmX _anth._	<1	2.08	<1	<1
9	Sm _seed_ + Sm _anth._	<1	72.8	<1	<1
10	SmF_seed_ + SmF _anth_.	<1	26.6	<1	<1
	Total FB2 conc. in each cultivar	144	108.5	16.3	8.1

All plants in all treatments except for the control were inoculated with *F. graminearum* within an 8 h window after the application of the BCA treatment. Concentrations at the Limit of Quantification LOQ < 16 were first divided by 2 for an approximate value, which was used in the calculation of total FB2 concentration (µg·kg^−1^). The treatments used were: (1) Control, no biocontrol agents (BCAs) or any other treatment; (2) Sm _seed_-BCA-*S. mycoparasitica* applied on the seed; (3) SmF _seed_-*S. mycoparasitica* + Fungicide on the seed; (4) Sm _anth_.-*S. mycoparasitica* applied at anthesis; (5) SmF _anth._-*S. mycoparasitica* + Fungicide at anthesis; (6) F.gr. _anth_.- a mixture of *F. graminearum* applied at anthesis; (7) SmX _seed_-*S. mycoparasitica* SMCD 2220-01 strain + *S. mycoparasitica* SMCD 2220-02(5) applied on seed; (8) SmX _anth_- *S. mycoparasitica* SMCD 2220-01 strain + *S. mycoparasitica* SMCD 2220-02(5) applied at anthesis; (9) Sm _seed_ + Sm _anth_-*S. mycoparasitica* seed + *S. mycoparasitica* at anthesis; and (10) SmF _seed_ + SmF _anth-_*S. mycoparasitica* + Fungicide seed and *S. mycoparasitica* + Fungicide at anthesis.

**Table 6:** Effect of plant cultivar and treatment on *Fusarium* community structure assessed with permutational multivariate analysis of variance (PERMANOVA).

Fusarium			
Source	d.f	F	*p*
Cultivar	3	0.95	0.51
Treatment	4	0.19	0.08

**Table 7:** Effect of plant cultivar and treatment on *Fusarium* community structure assessed with permutational multivariate analysis of variance (PERMANOVA). Permanova analysis indicated a tendency (*p* = 0.08) of treatment effect on *Fusarium* communities and associated mycotoxins.

Fusarium			
Source	d.f	F	*p*
Cultivar	3	0.95	0.51
Treatment	4	1.5	0.08

## Data Availability

Not applicable.
